# Abdominal pain due to the spinothalamic tract injury in patients with mild traumatic brain injury: a case report

**DOI:** 10.1186/s12883-020-01695-3

**Published:** 2020-04-02

**Authors:** Sung Ho Jang, Young Hyeon Kwon, Sung Jun Lee

**Affiliations:** Department of Physical Medicine and Rehabilitation, College of Medicine, Yeungnam University 317-1, Daemyungdong, Namku, Taegu, 705-717 Republic of Korea

**Keywords:** Diffusion tensor tractography, Spinothalamic tract, Traumatic axonal injury, Head trauma, Brain injury

## Abstract

**Background:**

We report on a patient with a mild traumatic brain injury (TBI) who developed abdominal pain due to spinothalamic tract (STT) injuries revealed by diffusion tensor tractography (DTT).

**Case presentation:**

A 53-year-old female patient suffered head trauma resulting from a backward fall. While bathing at a public bathhouse, she fell backward and struck the occipital area of her head against the floor. After the head trauma, she experienced pain in the abdomen and in both hands and feet. She underwent evaluations including conventional brain MRI, abdominal and pelvic ultrasonography, and stomach and intestine endoscopy. No abnormality was observed in her brain or abdomen. In addition, her abdominal pain had not been relieved by medical management. When she came to our hospital 4 years after the head trauma, her pain characteristics and severity were as follows: intermittent pain without allodynia or hyperalgesia; squeezing and warm creeping-like pain in the abdomen (visual analog scale score: 7); tingling pain in both hands and feet (visual analog scale score: 7). She was prescribed pregabalin and gabapentin, and her abdominal and limb pain was well-controlled at a tolerable level. On DTT 4 years after head trauma, the upper portion of the spinothalamic tracts (STTs) in both hemispheres showed partial tearing.

**Discussion and conclusions:**

Injury of the STT was demonstrated by using DTT in a patient who showed abdominal pain that was refractory to medical management following mild TBI. Our results suggest that central pain due to STT injury might be suspected in patients with abdominal pain that is refractory to medical management following TBI.

## Background

Central pain with characteristics of neuropathic pain is caused by injury of the central nervous system [1]. It has been reported that approximately 69% of patients with mild traumatic brain injury (TBI) experience central pain due to injury of the spinothalamic tract (STT) [2]. Because the management strategy for and the prognosis of central pain differ significantly from those for other pains ascribed to other pathophysiological etiologies, the elucidation of the pathophysiology of central pain is clinically important for patients with TBI [2].

The recent development of diffusion tensor tractography (DTT), images of which are reconstructed from diffusion tensor imaging (DTI) data, allows three-dimensional visualization and estimation of the STT [3]. Many studies using DTT have reported that injury of the STT is related to the pathophysiology of central pain in mild TBI [2–9]. Most of these studies have reported on central pain of the arm and leg. Conversely, no study on abdominal pain due to the STT injury has been reported.

In the current study, we report on a patient with mild TBI who developed abdominal pain. Results of DTT revealed that the patient had an injury of the STT.

## Case presentation

**Fig. 1:**
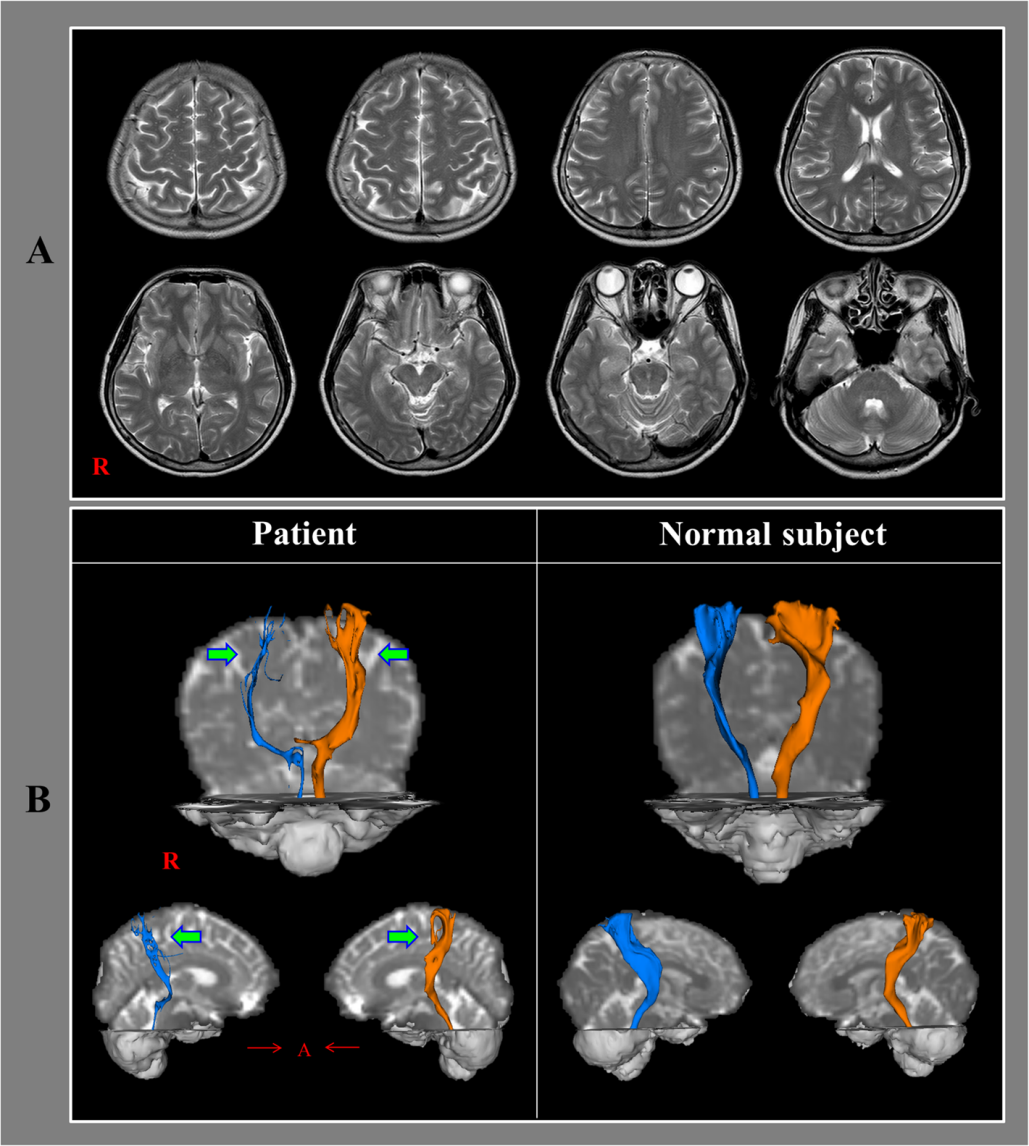
**a** T2-weighted brain magnetic resonance images taken at 4 years after onset shows no definite lesion. **b** Results of four-year post-trauma diffusion tensor tractography. In both hemispheres, the spinothalamic tract (STT) shows partial tearing in the upper portion (arrows) compared with those in a normal control (51-year-old female)

A 53-year-old female patient suffered a head trauma from a backward fall. While bathing at a public bathhouse, she fell backward and struck the occipital area of her head against the floor. The patient mentioned that she experienced loss of consciousness and post-traumatic amnesia for approximately 30 min after the accident. Her Glasgow Coma Scale was 15 when she arrived at the hospital [3, 10]. After the head trauma, she experienced pain in the abdomen and in both hands and feet as well as memory impairment, dysarthria, depressive mood, and auditory impairment which are common symptoms of TBI. She visited several hospitals and underwent various evaluations including conventional brain magnetic resonance imaging (MRI), abdominal and pelvic ultrasonography, and stomach and intestine endoscopy. No abnormalities were observed in her brain, abdomen, and pelvic organ. In addition, her abdominal pain had not been relieved by any medical management mainly at the department of internal medicine. Four years after the head trauma, she came to the rehabilitation department of our university hospital for evaluation of her brain. No specific lesion was observed on brain MRI that included T1-weighted, T2-weighted, and fluid-attenuated inversion recovery [FLAIR] images (Fig. 1a). Electromyography, nerve conduction and motor evoked potential studies for both upper and lower extremities, and paraspinal muscles did not show any abnormality. The characteristics and severity of her pain were assessed as follows: intermittent pain without allodynia or hyperalgesia; squeezing and warm creeping-like pain in the abdomen (visual analog scale score: 7); tingling pain in both hands and feet (visual analog scale score: 7). She notified that her pain was a little decreased with the passage of time from onset [11]. She was prescribed pregabalin (150 mg/day) and gabapentin (300 mg/day), and her abdominal and limb pain was well-controlled at a tolerable level. The patient provided signed, informed consent, and our institutional review board approved the study protocol.

### Diffusion tensor Tractography

DTI data were acquired 4 years after the patient’s head trauma by using a six-channel head coil on a 1.5 T Philips Gyroscan Intera (Philips, Best, Netherlands). For each of the 32 non-collinear diffusion sensitizing gradients, 70 contiguous slices were acquired parallel to the anterior commissure-posterior commissure line. Imaging parameters were as follows: acquisition matrix = 96 × 96; reconstructed matrix = 192 × 192; field of view = 240 × 240 mm^2^; TR = 10,726 ms; TE = 76 ms; parallel imaging reduction factor (SENSE factor) = 2; EPI factor = 49; b = 1000 s/mm^2^; NEX = 1; and slice thickness = 2.5 mm with no gap. For assessment of the STT, the Oxford Centre for Functional Magnetic Resonance Imaging of the Brain (FMRIB) Software Library (www.fmrib.ox.ac.uk/fsl) was used for analysis of DTI data. Affine multi-scale two-dimensional registration was used for correction of head motion effects and image distortion due to eddy currents. Fiber tracking was performed by using a probabilistic tractography method based on a multi-fiber model and was applied in the current study by utilizing tractography routines implemented in FMRIB Diffusion software (5000 streamline samples, 0.5 mm step lengths, curvature thresholds = 0.2). The STT was reconstructed by selecting fibers passing through regions of interest (ROI). To assess the STT, the seed ROI was placed in the posterolateral medulla on an axial slice, and two target ROIs with the option of termination were placed on a portion of the ventro-postero-lateral nucleus of the thalamus and primary somatosensory cortex [3]. The threshold values of two streamlines were applied for the results of fiber tracking for each tract.

On four-year post-trauma DTT, the upper portion of the STT in both hemispheres showed partial tearing (Fig. 1b).

## Discussion and conclusions

In this study, the patient experienced abdominal and limb pain after a head trauma. However, the precise cause of her pain had not been determined even though she had visited several hospitals and underwent various evaluations of her brain and abdomen. Partial STT injuries (i.e., partial tearing) in both hemispheres were observed on four-year DTT. Her abdominal and limb pain were well-controlled by administration of specific drugs (pregabalin and gabapentin) for neuropathic pain, which was not relieved by medical management [12]. As a result, her pain appeared to be related to the STT injury. No definite brain lesion was detected on conventional brain MRI, but injury of both STTs in the brain appeared to be the cause of the central pain; thus, traumatic axonal injury was the most likely pathogenetic mechanism for the STT injuries [10, 13–15].

In summary, by using DTT, STT injury was demonstrated in a patient who showed abdominal pain that was refractory to any medical management following mild TBI. Our results suggest that central pain from STT injury might be suspected in patients with abdominal pain that is not relieved by medical management following TBI. As a result, evaluation of the STT using DTT would be useful in patients who reveal unexplained and refractory abdominal pain following TBI. However, because this is a case report, the results have limited generalizability. Therefore, further studies that include larger numbers of cases are warranted. In addition, the limitations of DTT should be considered because regions of fiber complexity and fiber crossing can prevent full reflection of the underlying fiber architecture by DTT; thus, DTT may underestimate the fibers of the neural tracts [16].

## Data Availability

The datasets used and/or analysed during the current case reports are available from the corresponding author on reasonable request.

## Abbreviations

TBI: Traumatic brain injury
STT: Spinothalamic tract
DTT: Diffusion tensor tractography
DTI: Diffusion tensor imaging
MRI: Magnetic resonance imaging
FMRIB: Functional Magnetic Resonance Imaging of the Brain
ROI: Regions of interest
